# Colony fingerprint for discrimination of microbial species based on lensless imaging of microcolonies

**DOI:** 10.1371/journal.pone.0174723

**Published:** 2017-04-03

**Authors:** Yoshiaki Maeda, Hironori Dobashi, Yui Sugiyama, Tatsuya Saeki, Tae-kyu Lim, Manabu Harada, Tadashi Matsunaga, Tomoko Yoshino, Tsuyoshi Tanaka

**Affiliations:** 1 Division of Biotechnology and Life Science, Institute of Engineering, Tokyo University of Agriculture and Technology, Tokyo, Japan; 2 Malcom Co., Ltd., Tokyo, Japan; Pusan National University, REPUBLIC OF KOREA

## Abstract

Detection and identification of microbial species are crucial in a wide range of industries, including production of beverages, foods, cosmetics, and pharmaceuticals. Traditionally, colony formation and its morphological analysis (*e*.*g*., size, shape, and color) with a naked eye have been employed for this purpose. However, such a conventional method is time consuming, labor intensive, and not very reproducible. To overcome these problems, we propose a novel method that detects microcolonies (diameter 10–500 μm) using a lensless imaging system. When comparing colony images of five microorganisms from different genera (*Escherichia coli*, *Salmonella enterica*, *Pseudomonas aeruginosa*, *Staphylococcus aureus*, and *Candida albicans*), the images showed obvious different features. Being closely related species, *St*. *aureus* and *St*. *epidermidis* resembled each other, but the imaging analysis could extract substantial information (colony fingerprints) including the morphological and physiological features, and linear discriminant analysis of the colony fingerprints distinguished these two species with 100% of accuracy. Because this system may offer many advantages such as high-throughput testing, lower costs, more compact equipment, and ease of automation, it holds promise for microbial detection and identification in various academic and industrial areas.

## Introduction

Identification of microbial species is routinely performed in a wide range of industries, including production of beverages, foods, cosmetics, and pharmaceuticals. It is also of great importance in clinical diagnosis. A number of methods based on phenotypic and genotypic analyses have been proposed for microbial identification at different classification levels (*e*.*g*., family, genus, species, and strain). A typical phenotypic analysis is the comprehensive profiling of biochemical metabolic pathways for which several tool kits enabling rapid identification are commercially available (*e*.*g*., API series [1], BIOLOG [2], and VITEK 2 [3]). Mass spectrometry-based phenotypic analysis has been increasingly used for microbial identification, where whole microbial cells are directly subjected to matrix-assisted laser desorption/ionization time-of-flight mass spectrometry (MALDI-TOF-MS) [4]. On the other hand, genotypic analyses identify target microbes on the basis of their genomic sequences. Sequencing of 16S (for prokaryotes) or 18S (for eukaryotes) ribosomal DNA (rDNA) is the most common method for estimation of microbial species. Other methods such as genome hybridization and ribotyping can also accurately identify microbes at species/strain levels. Nevertheless, the phenotypic and genotypic methods mentioned above still require expensive reagents (*e*.*g*., polymerase, restriction enzymes, fluorophores, and chromophores), high expertise, and long assay duration.

For simpler microbial typing, Banada et al. proposed an optical method for identification and discrimination of bacterial species that is free from expensive reagents [5]. This method, referred to as BActerial Rapid Detection using Optical scatter Technology (BARDOT), involves irradiation of bacterial colonies grown in a Petri dish with a red laser to generate light scattering patterns. The light scattering patterns are dependent on the three-dimensional (3D) morphology of bacterial colonies, and the image analysis allows for species or even strain level discrimination. The BARDOT has distinguished *Listeria*, *Staphylococcus*, *Salmonella*, *Vibrio*, and *Escherichia* with classification accuracy of 90–99% [6]. Five species of *Listeria* [5], three species of *Vibrio* [7], and seven serogroups of *E*. *coli* [8] have been discriminated with the accuracy of >91%, >96%, and >81%, respectively. Although microbial discrimination based on colony-derived light scattering patterns is promising due to its high classification accuracy, the BARDOT usually analyzes colonies as large as 1.2–1.5 mm in diameter [5], and therefore long incubation time prior to laser irradiation limits the assay throughput. A new BARDOT system analyzing microcolonies with the diameter range of 100–200 μm was reported [9]. However, it only indicated the possibility to distinguish three different genera (*Listeria monocytogenes*, *E*. *coli*, and *Sa*. *enterica*), and further research is needed to extend this new BARDOT system to other species (*e*.*g*., discrimination of closely related species). Additionally, the BARDOT needs complex and bulky instruments including a laser, dish holder, and actuators for scanning large Petri dishes in order to analyze a number of colonies distributed there. For convenience with on-site use, miniaturized and inexpensive instrument setup is desired. Furthermore, the BARDOT differentiates colony on the basis of snapshot images, and thus time-dependent factors such as the growth rate and pattern variations are ignored.

To address these issues, in this study, we analyzed microbial colony formation with lensless imaging, and attempted to differentiate microbial species using the acquired images. Lensless imaging is an emerging technology for examination of biological objects without conventional microscopes [10–17]. Two-dimensional (2D) imaging photosensors (*e*.*g*., charge-coupled device, CCD; and complementary metal oxide semiconductor, CMOS) are used as imaging devices and objects above the sensor are directly projected. The lensless imaging system only needs the 2D imaging photosensor and a light source (*e*.*g*., light emitting diode), and thus it is simpler than other optical systems that have been employed for bacterial identification such as BARDOT, holographic microscopy [18, 19], angle resolved dark-field imaging [20]. A striking feature of this system is a wide imaging field as large as 2D sensors (mm^2^ scale). When microscopy is used to examine multiple objects distributed within a wide area on the mm^2^ scale, images are captured with scanning across the whole examination area, followed by image combining. In contrast, lensless imaging can obtain images on the mm^2^ scale within a second [14]. Because the scanning process is not required, rapid image capturing is possible. Image equipment setup requires only a 2D imaging sensor, leading to a simple, space-saving, inexpensive imaging system. Due to these advantages, lensless imaging systems have been used for various purposes such as detection of bacterial colonies [21] and single cell motility [22], classification of leukocytes into three main subtypes (lymphocytes, monocytes, and granulocytes) [23], and 3D tracking of human sperm [24]. In our group, simple and rapid cell counting platforms capable of imaging thousands of individual cells were developed [15]. Nonetheless, thus far, there are no studies that differentiate microbial species with lensless images. In this study, colony growth of microorganisms was monitored with lensless imaging, and discrimination of microbial species was performed at the genus level (*E*. *coli*, *Sa*. *enterica*, *Pseudomonas aeruginosa*, *St*. *aureus*, and *Candida albicans*) and species level (*St*. *aureus* and *St*. *epidermidis*). We extracted a number of discrimination parameters (referred to as a “colony fingerprint” in this article) by image analysis, and discrimination of microbial colonies was attempted by multivariate analysis of the colony fingerprint.

## Materials and methods

### Bacterial strains

*E*. *coli* strain ATCC 8739, *St*. *aureus* ATCC 6538, *St*. *epidermidis* ATCC 14990, *P*. *aeruginosa* ATCC 9027, *Sa*. *enterica* NBRC 100797, and *C*. *albicans* ATCC 10231 were used in this study. Usually, the genus names of microorganisms are abbreviates with one capital letter according to general nomenclature (*e*.*g*., *S*. *enterica* for *Salmonella enterica*, and *S*. *aureus* for *Staphylococcus aureus*). However, in this study, *Salmonella* and *Staphylococcus* are abbreviated with two letters, *Sa*. and *St*., respectively, to avoid confusion. Prior to colony formation testing, microorganisms were incubated in the lysogeny broth (LB) medium (5 ml) with shacking at 37°C overnight. Subsequently, the cell concentration was determined by cell counting using a microscope and hemocytometer.

### Setup and observation

**Fig 1:**
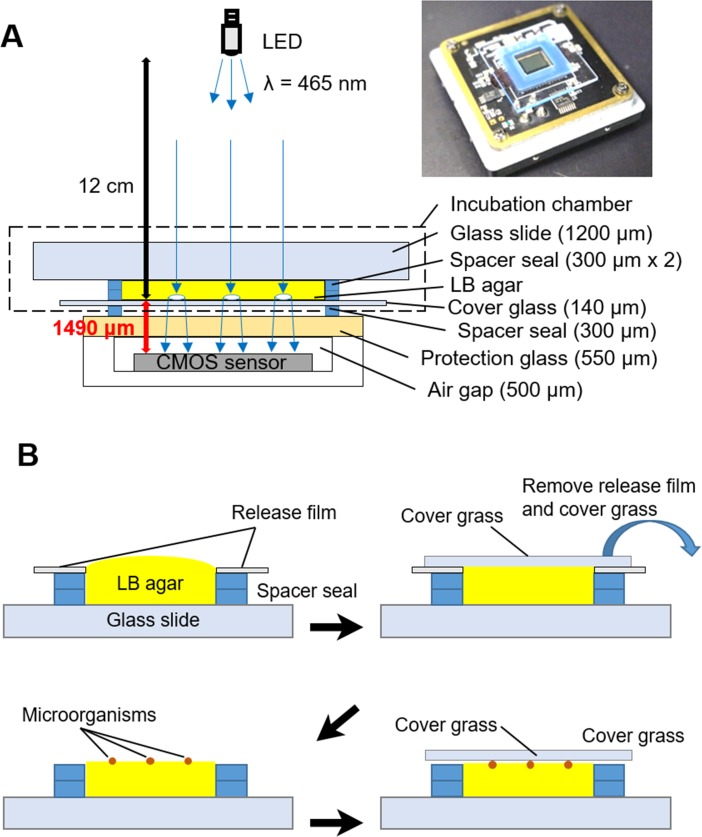
The lensless microcolony imaging system developed in this study. (A) A schematic diagram and photograph of the imaging system. Thickness of each component is described. (B) The schematic procedure of preparation of the incubation chamber.

A CMOS sensor composed of 2048 × 1536 pixels (pixel size: 3.2 μm, imaging area: 6.55 × 4.92 mm^2^, DFK61BUC02, The Imaging Source Europe GmbH, Bremen, Germany) was used for imaging. The incubation microchamber composed of a glass slide, two spacer seals (9 × 9 mm^2^, thickness 300 μm each) and a cover glass was directly mounted on the protection glass of the CMOS image sensor (Fig 1A). Bacterial colony formation in the incubation chamber was assayed as described below (Fig 1B). The incubation chamber without a cover glass was fulfilled with 1.5% (w/v) agarose-LB medium in the liquid state. At this point, a sticky side of the spacer seal was covered by a release film. A cover glass was mounted to make the surface of LB-agar flat. After 20 min, LB-agar was solidified, and then the cover glass and the release film covering the spacer seal were carefully removed. Bacterial suspension (3.2 × 10^5^ cells/ml, 1 μl) was dropped on the LB-agar. A cover glass was mounted on the LB-agar again. The cover glass and spacer seal were tightly agglutinant to prevent water evaporation. A blue light-emitting diode (LED) was located 12 cm above the sensor and illuminates the chamber. A blue LED was employed because light scattering effect determines the contrast of the colony images projected on the CMOS sensor. Light with a shorter wavelength is expected to be scattered by colonies more strongly than that with longer wavelength, and it could result in higher contrast image of the colonies against the background. The developed lensless imaging system composed of CMOS sensor, chamber, and LED was kept at 37°C (S1 Fig). Images were automatically captured every 5 min (exposure time: 1/18 sec) under the control of the IC Capture 2.2 software (The Imaging Source Europe GmbH, Bremen, Germany). Conventional microscopic observation (BX61, OLYMPUS, Japan) was performed on the identical area that the lensless imaging system captured. We repeated the independent culture experiments twice for each microbial species using different set of the LB-ager chamber and CMOS sensor, and randomly selected colonies from the two monitoring data for the following analyses.

### Image processing

The image analysis described below was performed by using ImageJ [25] and MATLAB (The MathWorks, Inc., Massachusetts, USA). First, the contrast of original lensless images was enhanced by remapping the data values to fill the entire intensity range of (0, 255) using the auto-adjusting function for intensity values in MATLAB. Subsequently, each pixel value is subtracted from the maximum pixel value, and the difference is used as the pixel value in the output images; *i*.*e*., black-and-white balance is inverted. Then, the images were binarized with Otsu’s thresholding method [26] by which the pixels were represented in 10 gray levels, and dichotomized into two classes (*i*.*e*., background and colony regions) with a threshold at the maximum level. After the binarization, fill-up processing was executed to determine colony regions. Finally, discriminative parameters (histogram deviation, G; donutness, D; entropy, H; and energy density, E), which are described in detail below, were calculated from the contrast-adjusted and inverted lensless images at the colony regions.

### Discriminative parameters and cluster analysis

For discrimination of microbial colonies, 7 parameters—maximum specific growth rate (μ_max_), colony appearance time (t_a_), average intensity (I), histogram deviation (G), donutness (D), entropy (H), and energy density (E)—were extracted as a “colony fingerprint” in this study. The colonies were selected randomly unless they did not merge during the imaging period. Specific growth rates (μ) of microbial colonies were calculated by means of the following formula: μ = ln(At—A_t-1_)/{t—(t—1)}, where t is incubation time (hours), and A_t_ is the area of a colony region after t hours of incubation. It should be noted that A_t_ was measured every 1 hour, and μ_max_ is defined as the maximum value of μ within 10 hours. To determine the colony appearance time (t_a_), signal intensity profile was analyzed along lines (100 pixels) which passes through a colony region or a background region. Differences between the maximal and minimal intensities on the lines passing through a colony region and background are defined as S and N, respectively. It should be noted that S and N were measured every hour, and t_a_ is defined as the minimum time point when S/N exceeds 2. Relative intensity (I) is calculated based on the following formula, I = I_c_—I_b_, where I_c_ and I_b_ are the means of intensity (ranging from 0 to 255) in a colony region, and background region (a square 100 × 100 pixels), respectively. The parameters mentioned above were determined by means of ImageJ. The parameters mentioned hereinafter were determined by MATLAB software with colony images whose diameter was approximately 250 μm. To determine the histogram deviation (G), the brightness value histogram of a colony region with 20 bins was constructed. G is defined as a standard deviation value of the pixel numbers included in each bin, and indicates narrowness of intensity distribution. To determine the donutness (D), averaged relative intensities of a whole colony region (D_w_) and its central region with half diameter (D_c_) was calculated. D is defined as the ratio D_w_/D_c_. Entropy (H) and energy density (E) represent regularity of intensity distribution and periodicity of scatter patterns in a colony region, and were determined by entropy and energy functions in MATLAB, respectively. Principal component analysis (PCA), k-means clustering, and linear discriminant analysis (LDA) were performed using R 3.1.2 (R Foundation for Statistical Computing, Vienna, Austria). In general, PCA is a mathematical process to reduce the dimensionality of the data while retaining most of the variation in the data set [27]. Contribution ratios indicate how much percentage of whole tendency of the data set is presented by each PC.

## Results

### Lensless imaging of microbial colonies

**Fig 2:**
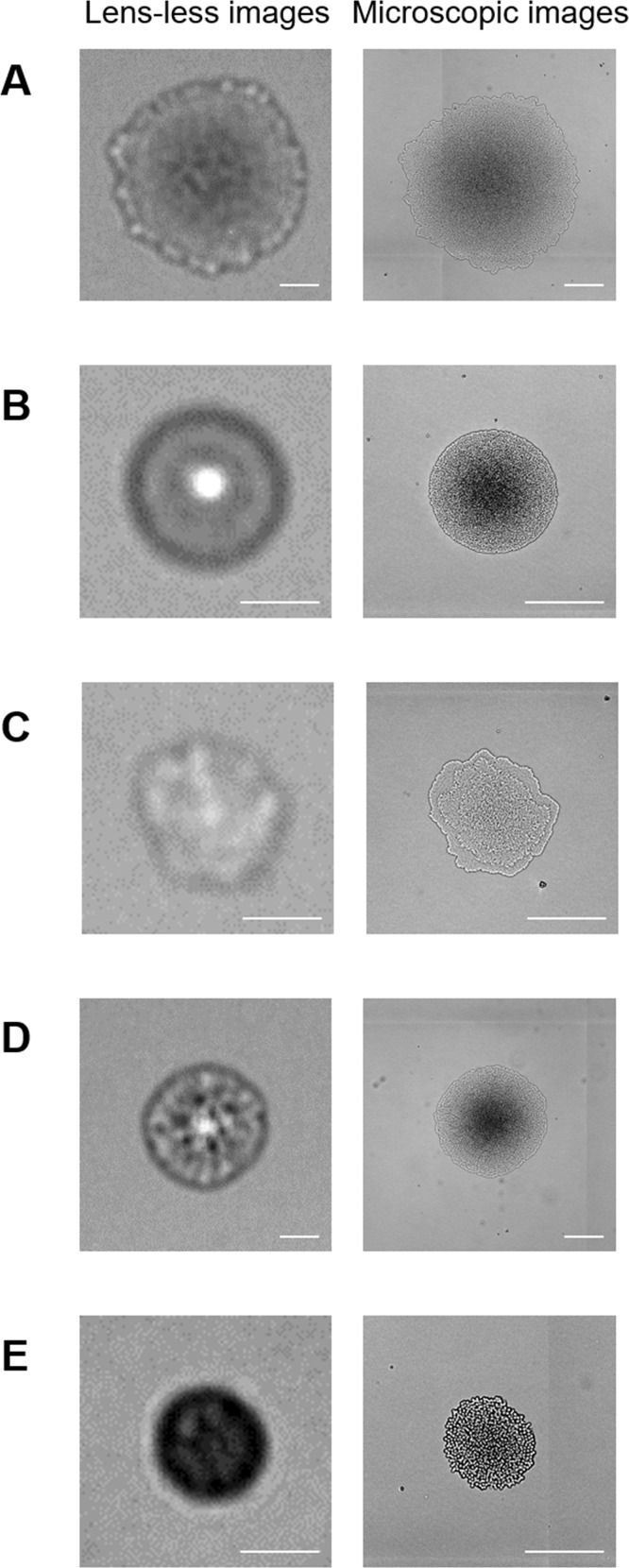
**Images of colonies *of* (A) *E*. *coli*, (B) *St*. *aureus*, (C) *P*. *aeruginosa*, (D) *Sa*. *enterica*, *and* (E) *C*. *albicans* acquired by the lensless imaging system and a microscope after 8 hours of incubation.** Scale bar = 100 μm.

The CMOS sensors can capture images as wide as the size of its detection area (6.55 × 4.92 mm^2^, corresponding to ~55 images of microscopic observation at 100× magnification; S2 Fig) within a second. When *E*. *coli* was examined, multiple colonies were clearly visualized in a single image (S2 Fig). It is likely that the colony size can be affected by the density populations; colonies in the high colony density area tend to be small in size. This could be caused by the combination effects of the limitation of the nutrition supply from LB-agar media and intercellular communications between bacteria such as quorum sensing. The colonies were selected randomly from the lensless images regardless of the colony density, and subjected to subsequent analyses. Fig 2 shows the images of microbial colonies (*E*. *coli*, *St*. *aureus*, *P*. *aeruginosa*, *Sa*. *enterica*, *and C*. *albicans*) examined by lensless imaging and microscopy. Each microorganism exhibited specific patterns in the lensless images. Lensless images of *E*. *coli* colonies have a central dark zone surrounded by an outermost frill. *St*. *aureus* colonies have a round shape with a bright spot in their central area. *P*. *aeruginosa* colonies are of indefinite shape, and the image contrast is lower than that of other microorganisms tested in this study. *Sa*. *enterica* colonies have a round shape with random texture. *C*. *albicans* colonies show a round shape and a dark pattern.

It was found that microbial colonies observed with lensless imaging were larger than with microscopy. To assess this finding, 20 *E*. *coli* colonies of different sizes were analyzed by both systems and compared. As a result, the sizes of *E*. *coli* colonies analyzed by lensless imaging and with the microscope have a proportional relation with coefficient of 1.145, indicating that the lensless imaging system overestimated the colony size as compared to microscopy (S3 Fig).

The lensless-imaging system developed in this study allowed us to demonstrate time-lapse imaging of microbial colonies (S1–S5 Movies and S4 Fig). Colony growth was quantitatively expressible as an expansion of colony region areas (S5 Fig). Appearance time (t_a_, definition is described in the Materials and Methods section) of *E*. *coli*, *St*. *aureus*, *P*. *aeruginosa*, *Sa*. *enterica*, *and C*. *albicans* was 2, 4–5, 4, 3, and 0 h, respectively. *C*. *albicans* cells were detectable without incubation on the lensless-imaging system due to the large size of the eukaryotic cells, although the obtained images of the cells did not clearly outline the shape of the cells in contrast to microscopy (S6 Fig). Immediately after plating, *E*. *coli* cells, whose size was found to be 2.39 ± 1.12 μm and 1.19 ± 0.17 μm in length and width according to microscopic examination were shown to be well dispersed as stand-alone cells according to microscopic examination, and undetectable by lensless imaging (S6A Fig). In contrast, some of *C*. *albicans* cells (length and width were 8.69 ± 3.96 and 5.26 ± 0.85 μm, respectively) were in a single-cell state, while others were in an aggregated state; both of them were detectable as circular objects by the lensless-imaging system (S6B Fig). This finding makes sense because spatial resolution of the images is limited to twice the pixel size of the CMOS sensor (the pixel size is 3.2 μm) according to the Nyquist criterion.

### Discrimination of microbes belonging to different genera

**Fig 3:**
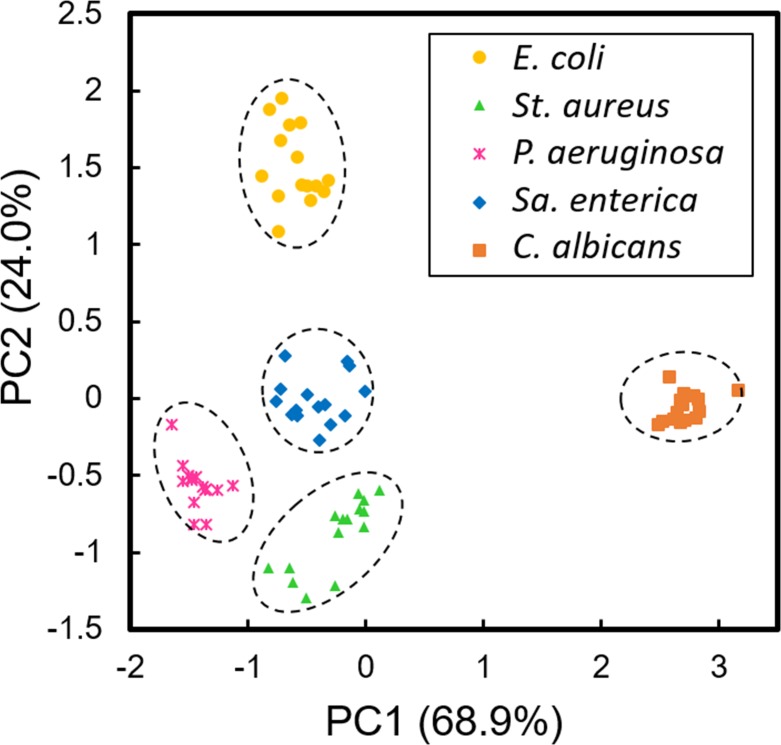
Discrimination results on *E*. *coli*, *St*. *aureus*, *P*. *aeruginosa*, *Sa*. *enterica*, and *C*. *albicans* based on lensless images. Principal component analysis (PCA) of maximum specific growth rate (μ_max_), colony appearance time (t_a_), and relative intensity (I) extracted from lensless images of 15 microcolonies of 5 microbes. Dashed circles represent the clusters generated by k-means cluster analysis. Data points of 15 colonies of the each microbial species were obtained from two independent culture experiments using different set of the LB-ager chamber and CMOS sensor. Contribution ratios (%) of PC1 and PC2 are shown, respectively.

We extracted three discrimination parameters, the maximum specific growth rate (μ_max_), colony appearance time (t_a_), and relative intensity (I) from lensless images of 15 colonies of 5 microbial species described above. Fig 3 shows principal component analysis (PCA) of the 75 colony fingerprint vectors (15 colonies of 5 microbial species) composed of the 3 parameters, and different species were plotted by different marks and colors, which were obviously separated. When the 75 vectors were classified into five clusters by k-means clustering, all five clusters were composed of 15 vectors derived from identical microbes.

To confirm the reproducibility of this system, we completely repeated the same experiment. Prior to the repeated experiment, completely new bacterial cultures were prepared, and whole process including culture preparation, colony selection, image analysis was repeated by the operator different from the one who conducted the experiment shown in Fig 3. As a result, the repeated experiment was able to classify the 5 microbes belonging to different genera (*E*. *coli*, *St*. *aureus*, *P*. *aeruginosa*, *Sa*. *enterica*, and *C*. *albicans*) with 100% accuracy (S7 Fig). This result is identical with the experiment previously performed. Therefore, this system could distinguish the microbes in different genera with high reproducibility.

### Discrimination of *Staphylococcus aureus* and *Staphylococcus epidermidis*

**Fig 4:**
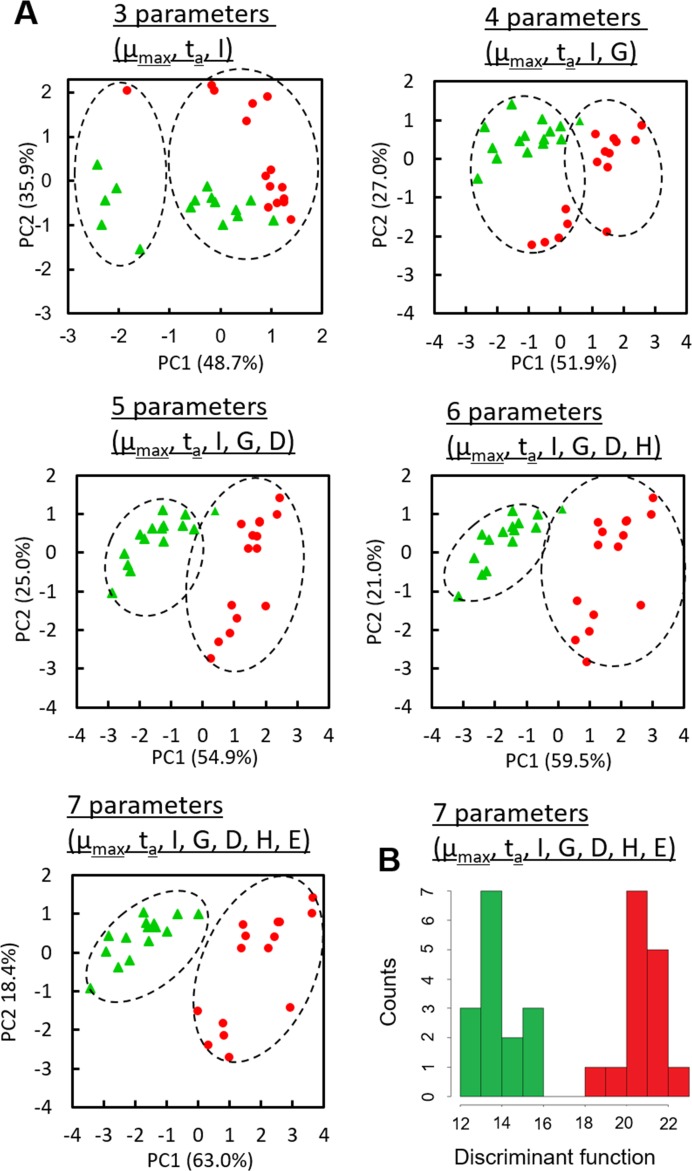
Discrimination results on *St*. *aureus* and *St*. *epidermidis* based on lensless images. Data points of 15 colonies of the each microbial species were obtained from two independent culture experiments using different set of the LB-ager chamber and CMOS sensor. Contribution ratios (%) of PC1 and PC2 are shown, respectively. (A) Principal component analysis (PCA) of maximum specific growth rate (μ_max_), colony appearance time (t_a_), relative intensity (I), histogram deviation (G), donutness (D), entropy (H), and energy density (E) extracted from lensless images of 15 microcolonies of *St*. *aureus* and *St*. *epidermidis*. Dashed circles represent the clusters generated with k-means cluster analysis. (B) A histogram of the value of discriminant function (F) based on linear discriminant analysis. All of the F values of *St*. *aureus* and *St*. *epidermidis* were less than 16 (green columns) and more than 18, respectively (red columns).

To test whether lensless-imaging based bacterial discrimination can distinguish closely related bacteria *St*. *aureus* and *St*. *epidermidis* [28], various parameters, *i*.*e*., the maximum specific growth rate (μ_max_), colony appearance time (t_a_), relative intensity (I), histogram deviation (G), donutness (D), entropy (H), and energy density (E), were extracted from lensless images of 15 colonies of each *Staphylococcus*, which resemble one another to the naked eye (S4B and S4F Fig). Fig 4A shows PCA of the 30 vectors (15 colonies of *St*. *aureus* and *St*. *epidermidis*) composed of the 3–7 parameters, and different species were plotted by different marks and colors. In contrast to the case of the five microbes belonging to different genera, the colony fingerprint vectors composed of μ_max_, t_a_, and I were not clearly separated. By increasing parameter numbers, separation of two clusters became clearer. The 30 vectors were classified into 2 clusters by k-means clustering with the increasing parameter numbers. When all of the 7 parameters were used, both clusters were separated with the constituent 15 colony fingerprint vectors derived from identical microbes. In order to discriminate the bacterial species by lensless imaging, linear discriminant analysis (LDA) was performed to determine the discriminant function (F) as shown below

$$F=\left(13.12316\right)\times \mu_{max}+\left(\text{-}0.2817895\right)\times t_{a}+\left(0.6712573\right)\times I+\left(0.02388584\right)\times G\left(\text{-}9.171612\right)\times D+\left(2.809352\right)\times H+\left(\text{-}0.000000001247725\right)\times E$$ (1)

The values of the discrimination function were obviously separated between *St*. *aureus* and *St*. *epidermidis* (Fig 4B), and discrimination accuracy was 100%.

To confirm the reproducibility, we completely repeated the same experiment as mentioned above. As a result, the repeated experiment was able to classify the *St*. *aureus* and *St*. *epidermidis* by LDA with 100% accuracy (S8 Fig). This result also supported the reproducibility of this system.

## Discussion

The lensless imaging system with a 2D image sensor successfully visualized microbial colonies and their growth in a wide field of view. The size of colonies visualized by lensless imaging was proportional with that according to microscopy, while the lensless imaging overestimated the colony size approximately by 14.5% as compared to microscopy (S3 Fig). The overestimation of colony size by the lensless imaging could be caused by the effect of light diffraction from the outer edge of the colonies that are placed 1490 μm above the CMOS image sensor (Fig 1).

Microbial colonies tested in this study showed characteristic light scattering patterns. In particular, *E*. *coli* colonies were composed of a relatively dark center area and an outer bright periphery region (Fig 2A). This feature could represent the three-dimensional (3D) structure of *E*. *coli* colonies. Previously, confocal laser scanning microscopy revealed the colony growth dynamics of *E*. *coli* [29]. The bacterial cells organized into the circular microcolony consisting of monolayer cells at first, and the monolayer microcolony expanded outward. At a certain point, the colony growth transited from 2D expansion to 3D growth where two to multi-layers appeared on the bottom layer. Interestingly, the monolayer region remained at the outer ring of the circular colony, and the width of the monolayer was constant during the colony growth. Our microscopic image of the colony also represented the same feature (Fig 2A, right), and the bright peripheral region shown in the lensless image (Fig 2A, left) is likely to correspond to the monolayer region. This result suggests that the lensless imaging system can provide 3D information on microbial colonies. Since the colony morphology in 3D is related to inherent characters of microorganisms, the lensless imaging could be applicable to identification or discrimination of microbial species.

When microorganisms from different genera (*E*. *coli*, *St*. *aureus*, *P*. *aeruginosa*, *Sa*. *Enterica*, and *C*. *albicans*) were photographed, lensless images of the microcolonies were obviously different from each other (Fig 2). Colony fingerprint vectors composed of only 3 parameters (μ_max_, t_a_, and I) were clearly separated (Fig 3). Although lensless imaging of the colony growth and the PCA of the fingerprints extracted from the lensless images demonstrated the proof of concept of this study, it is true that the microscopic imaging could also discriminate these 5 species. Theoretically, it is possible to monitor the colony growth by microscopy, and perform the PCA based on the microscopic images. However, it is needed to obtain μ_max_ and t_a_ under the conditions suitable for microbial colony growth (*e*.*g*., 37°C). To do so, the LB-agar plates should be kept in an incubator at 37°C for colony growth, and at every observation time point, they should be ejected from the incubator and subjected to microscopy. This operation procedure is labor intensive and limits the analytical throughput, when we consider the practical applications (*e*.*g*., contamination check for production of beverages and foods). In contrast, the lensless imaging system consists of simple and compact set-ups (Fig 1). Several set-ups of the whole systems with incubation microchambers can be kept in a small incubator (S1 Fig), so that simultaneous multi-parallel analysis can be easily performed without transferring the samples between the incubator and observation system. Therefore, the lensless imaging system presented in this study should have the advantage of analytical throughput over microscopy.

As compared to the 5 species in different genera, lensless images of *St*. *aureus* and *St*. *epidermidis* much resembled each other. Indeed, the conventional discrimination method for these species is based on the enzyme (coagulase) activity assay because colony morphologies of these two are almost identical to the naked eye. We extracted more discrimination parameters, and performed LDA to compute the discriminant function (Eq 1). Given the coefficients in Eq 1, the maximum specific growth rate (μ_max_) mainly contributes to the discrimination, while donutness (D) and entropy (H) were also important parameters. *St*. *aureus* grew more slowly than *St*. *epidermidis* did, and the μ_max_ of those were 0.666 ± 0.034 and 0.806 ± 0.055, respectively. This difference in growth behavior was one of the critical factors for discrimination of these species, and lensless imaging can take advantage of real-time monitoring of the colony formation process to obtain growth-related parameters.

It is worth noting that clinically isolated microorganisms frequently have mutations which alter the growth behavior [30, 31]. Mutations in flagella and pili could also affect the colony size and morphology [32, 33]. These mutations potentially affect the discrimination performance, and thus it is preferable to correct the colony fingerprints from theses mutants and establish a large scale of colony fingerprint library. Commercially available MALDI-TOF MS-based bacterial identification systems contain MS libraries for several hundred species. The expansion of the library scale will allow us to apply the lensless imaging analysis for identification of microorganisms isolated from clinical and/or environmental samples.

In the present study, the microorganisms were cultured separately prior to extraction of colony fingerprints, and the fingerprints extracted from different microorganisms were shown to be distinguishable. The next challenge should be identification of the microorganisms existing in mixed population, and it will be investigated in the near future projects.

## Conclusions

We developed a lensless imaging system to examine microbial colonies. Prokaryotic and eukaryotic microbial colonies were successfully examined by means of the system, and each microorganism showed different colony patterns, which could represent the 3D structure of each colony. It is also possible to analyze colony growth over time. Furthermore, a number of quantitative parameters could be extracted from the lensless images of microbial colonies. Such parameters, referred to as colony fingerprints, helped us to discriminate microbial species. By taking advantage of a number of useful features of the wide observation area, easy handling, and small and inexpensive set-ups, lensless imaging could become a powerful tool of microorganism research.

## Supporting information

S1 Fig Six sets of lens-less imaging systems contained in the incubation cover. (TIF)

S2 Fig Lensless imaging of bacterial colonies. The entire image (sensing area: 6.55 × 4.92 mm) with *E*. *coli* colonies acquired by means of a CMOS sensor (A) and the corresponding microscopic image (B).
(TIF)

S3 Fig Correlation between *E*. *coli* colony size as measured by lensless imaging and microscopy. (TIF)

S4 Fig **Time lapse images of colonies of (A) *E*. *coli*, (B) *St*. *aureus*, (C) *P*. *aeruginosa*, (D) *Sa*. *enterica*, (E) *C*. *albicans*, and (F) *St*. *epidermidis* acquired by the lensless imaging system.** Scale bar = 100 μm.
(TIF)

S5 Fig **Colony size variation of (A) *E*. *coli*, (B) *St*. *aureus*, (C) *P*. *aeruginosa*, (D) *Sa*. *enterica*, (E) *C*. *albicans*, and (F) *St*. *epidermidis* measured in lensless images.** Lines (arbitrary colors) are plots for individual 15 colonies.
(TIF)

S6 Fig **Bright-field images of *E*. *coli* (A) and *C*. *albicans* (B) on LB agar.** The images were acquired by means of the lensless imaging system and microscope immediately after plating. Yellow circles on the lens-less image show *C*. *albicans* cells. Yellow arrows on the microscopic images show *E*. *coli* and *C*. *albicans* cells. Scale bar = 20 μm. Line profile analysis for the lensless image of *C*. *albicans* cells was performed (C).
(TIF)

S7 Fig Repeated experiment of discrimination of *E*. *coli*, *St*. *aureus*, *P*. *aeruginosa*, *Sa*. *enterica*, and *C*. *albicans* based on lensless images. Prior to the repeated experiment, completely new bacterial cultures were prepared, and whole process including culture preparation, colony selection, image analysis was repeated by the operator different from the one who conducted the experiment shown in Fig 3. Principal component analysis (PCA) of maximum specific growth rate (μ_max_), colony appearance time (t_a_), and relative intensity (I) extracted from lensless images of 15 microcolonies of 5 microbes. Contribution ratios (%) of PC1 and PC2 are shown, respectively. Dashed circles represent the clusters generated by k-means cluster analysis.
(TIF)

S8 Fig Repeated experiment of discrimination of *St*. *aureus* and *St*. *epidermidis* based on lensless images. Prior to this repeated experiment, completely new bacterial cultures were prepared, and whole process including culture preparation, colony selection, and image analysis was conducted by the operator different from the one who conducted the experiment shown in Fig 4. (A) Principal component analysis (PCA) of maximum specific growth rate (μ_max_), colony appearance time (t_a_), relative intensity (I), histogram deviation (G), donutness (D), entropy (H), and energy density (E) extracted from lensless images of 15 microcolonies of *St*. *aureus* and *St*. *epidermidis*. Contribution ratios (%) of PC1 and PC2 are shown, respectively. Dashed circles represent the clusters generated by k-means cluster analysis. (B) A histogram of the value of discriminant function (F) based on linear discriminant analysis.
(TIF)

S1 Movie Colony formation of *E*. *coli* monitored by lensless imaging. (AVI)

S2 Movie Colony formation of *St*. *aureus* monitored by lensless imaging. (AVI)

S3 Movie Colony formation of *P*. *aeruginosa* monitored by lensless imaging. (AVI)

S4 Movie Colony formation of *Sa*. *enterica* monitored by lensless imaging. (AVI)

S5 Movie Colony formation of *C*. *albicans* monitored by lensless imaging. (AVI)

## References

[1] Edinger RC, Migneault PC, Nolte FS. Supplementary rapid biochemical test panel for the API 20E bacterial identification system. J Clin Microbiol. 1985;22(6):1063–5. 3905846 10.1128/jcm.22.6.1063-1065.1985 PMC271883

[2] Klingler JM, Stowe RP, Obenhuber DC, Groves TO, Mishra SK, Pierson DL. Evaluation of the Biolog automated microbial identification system. Appl Environ Microbiol. 1992;58(6):2089–92. 11536500 10.1128/aem.58.6.2089-2092.1992 PMC195731

[3] Funke G, Monnet D, deBernardis C, von Graevenitz A, Freney J. Evaluation of the VITEK 2 system for rapid identification of medically relevant gram-negative rods. J Clin Microbiol. 1998;36(7):1948–52. 9650942 10.1128/jcm.36.7.1948-1952.1998 PMC104958

[4] Welker M, Moore ER. Applications of whole-cell matrix-assisted laser-desorption/ionization time-of-flight mass spectrometry in systematic microbiology. Syst Appl Microbiol. 2011;34(1):2–11. 10.1016/j.syapm.2010.11.013 21288677

[5] Banada PP, Guo S, Bayraktar B, Bae E, Rajwa B, Robinson JP, et al Optical forward-scattering for detection of *Listeria monocytogenes* and other *Listeria* species. Biosens Bioelectron. 2007;22(8):1664–71. 10.1016/j.bios.2006.07.028 16949268

[6] Banada PP, Huff K, Bae E, Rajwa B, Aroonnual A, Bayraktar B, et al Label-free detection of multiple bacterial pathogens using light-scattering sensor. Biosens Bioelectron. 2009;24(6):1685–92. 10.1016/j.bios.2008.08.053 18945607

[7] Huff K, Aroonnual A, Littlejohn AE, Rajwa B, Bae E, Banada PP, et al Light-scattering sensor for real-time identification of *Vibrio parahaemolyticus*, *Vibrio vulnificus* and *Vibrio cholerae* colonies on solid agar plate. Microb Biotechnol. 2012;5(5):607–20. Epub 2012/05/23. 10.1111/j.1751-7915.2012.00349.x 22613192 PMC3815873

[8] Tang Y, Kim H, Singh AK, Aroonnual A, Bae E, Rajwa B, et al Light scattering sensor for direct identification of colonies of *Escherichia coli* serogroups O26, O45, O103, O111, O121, O145 and O157. PLoS One. 2014;9(8):e105272 10.1371/journal.pone.0105272 25136836 PMC4138183

[9] Bae E, Bai N, Aroonnual A, Bhunia AK, Hirleman ED. Label-free identification of bacterial microcolonies via elastic scattering. Biotechnol Bioeng. 2011;108(3):637–44. 10.1002/bit.22980 21246511

[10] Ozcan A, Demirci U. Ultra wide-field lens-free monitoring of cells on-chip. Lab Chip. 2008;8(1):98–106. 10.1039/b713695a 18094767

[11] Zheng G, Lee SA, Antebi Y, Elowitz MB, Yang C. The ePetri dish, an on-chip cell imaging platform based on subpixel perspective sweeping microscopy (SPSM). Proc Natl Acad Sci U S A. 2011;108(41):16889–94. 10.1073/pnas.1110681108 21969539 PMC3193234

[12] Su TW, Seo S, Erlinger A, Ozcan A. High-throughput lensfree imaging and characterization of a heterogeneous cell solution on a chip. Biotechnol Bioeng. 2009;102(3):856–68. 10.1002/bit.22116 18853435 PMC4183348

[13] Jin G, Yoo IH, Pack SP, Yang JW, Ha UH, Paek SH, et al Lens-free shadow image based high-throughput continuous cell monitoring technique. Biosens Bioelectron. 2012;38(1):126–31. 10.1016/j.bios.2012.05.022 22664383

[14] Tanaka T, Saeki T, Sunaga Y, Matsunaga T. High-content analysis of single cells directly assembled on CMOS sensor based on color imaging. Biosens Bioelectron. 2010;26(4):1460–5. 10.1016/j.bios.2010.07.081 20728336

[15] Saeki T, Hosokawa M, Lim TK, Harada M, Matsunaga T, Tanaka T. Digital cell counting device integrated with a single-cell array. PLoS One. 2014;9(2):e89011 10.1371/journal.pone.0089011 24551208 PMC3923895

[16] Roy M, Seo D, Oh S, Chae Y, Nam MH, Seo S. Automated micro-object detection for mobile diagnostics using lens-free imaging technology. Diagnostics. 2016;6. 10.3390/diagnostics6020017 PMC4931412 27164146

[17] Roy M, Seo D, Oh CH, Nam MH, Kim YJ, Seo S. Low-cost telemedicine device performing cell and particle size measurement based on lens-free shadow imaging technology. Biosens Bioelectron. 2015;67:715–23. 10.1016/j.bios.2014.10.040 25459053

[18] Javidi B, Moon I, Yeom S, Carapezza E. Three-dimensional imaging and recognition of microorganism using single-exposure on-line (SEOL) digital holography. Opt Express. 2005;13(12):4492–506. 19495364 10.1364/opex.13.004492

[19] Jo Y, Jung J, Kim MH, Park H, Kang SJ, Park Y. Label-free identification of individual bacteria using Fourier transform light scattering. Opt Express. 2015;23(12):15792–805. 10.1364/OE.23.015792 26193558

[20] Wilson BK, Vigil GD. Automated bacterial identification by angle resolved dark-field imaging. Biomed Opt Express. 2013;4(9):1692–701. 10.1364/BOE.4.001692 24049690 PMC3771840

[21] Jung JH, Lee JE. Real-time bacterial microcolony counting using on-chip microscopy. Sci Rep. 2016;6:21473 10.1038/srep21473 26902822 PMC4763285

[22] Pushkarsky I, Liu Y, Weaver W, Su TW, Mudanyali O, Ozcan A, et al Automated single-cell motility analysis on a chip using lensfree microscopy. Sci Rep. 2014;4:4717 10.1038/srep04717 24739819 PMC3989554

[23] Vercruysse D, Dusa A, Stahl R, Vanmeerbeeck G, de Wijs K, Liu C, et al Three-part differential of unlabeled leukocytes with a compact lens-free imaging flow cytometer. Lab Chip. 2015;15(4):1123–32. 10.1039/c4lc01131g 25537881

[24] Su TW, Xue L, Ozcan A. High-throughput lensfree 3D tracking of human sperms reveals rare statistics of helical trajectories. Proc Natl Acad Sci U S A. 2012;109(40):16018–22. 10.1073/pnas.1212506109 22988076 PMC3479566

[25] Schneider CA, Rasband WS, Eliceiri KW. NIH Image to ImageJ: 25 years of image analysis. Nat Methods. 2012;9(7):671–5. 22930834 10.1038/nmeth.2089 PMC5554542

[26] Otsu N. A threshold selection method from gray-level histograms. Automatica. 1975;11(285–296):23–7.

[27] Ringner M. What is principal component analysis? Nat Biotechnol. 2008;26(3):303–4. 10.1038/nbt0308-303 18327243

[28] Kawamura Y, Hou XG, Sultana F, Hirose K, Miyake M, Shu SE, et al Distribution of *Staphylococcus* species among human clinical specimens and emended description of Staphylococcus caprae. J Clin Microbiol. 1998;36(7):2038–42. 9650958 10.1128/jcm.36.7.2038-2042.1998 PMC104974

[29] Su PT, Liao CT, Roan JR, Wang SH, Chiou A, Syu WJ. Bacterial colony from two-dimensional division to three-dimensional development. PLoS One. 2012;7(11):e48098 10.1371/journal.pone.0048098 23155376 PMC3498271

[30] Matsuo M, Hishinuma T, Katayama Y, Hiramatsu K. A mutation of RNA polymerase beta subunit (RpoC) converts heterogeneously vancomycin-intermediate *Staphylococcus aureus* (hVISA) into "slow VISA". Antimicrob Agents Chemother. 2015;59(7):4215–25. 10.1128/AAC.00135-15 25941225 PMC4468653

[31] Ishii K, Tabuchi F, Matsuo M, Tatsuno K, Sato T, Okazaki M, et al Phenotypic and genomic comparisons of highly vancomycin-resistant *Staphylococcus aureus* strains developed from multiple clinical MRSA strains by *in vitro* mutagenesis. Sci Rep. 2015;5:17092 10.1038/srep17092 26603341 PMC4658547

[32] Zhang S, McCormack FX, Levesque RC, O'Toole GA, Lau GW. The flagellum of *Pseudomonas aeruginosa* is required for resistance to clearance by surfactant protein A. PLoS One. 2007;2(6):e564 10.1371/journal.pone.0000564 17593964 PMC1891440

[33] Murray TS, Kazmierczak BI. *Pseudomonas aeruginosa* exhibits sliding motility in the absence of type IV pili and flagella. J Bacteriol. 2008;190(8):2700–8. 10.1128/JB.01620-07 18065549 PMC2293233

