# Supplementary figures and images for: Colony fingerprint for discrimination of microbial species based on lensless imaging of microcolonies
Source: PLoS One. 2017 Apr 3;12(4):e0174723. doi: 10.1371/journal.pone.0174723 (PMC5378366; doi:10.1371/journal.pone.0174723)

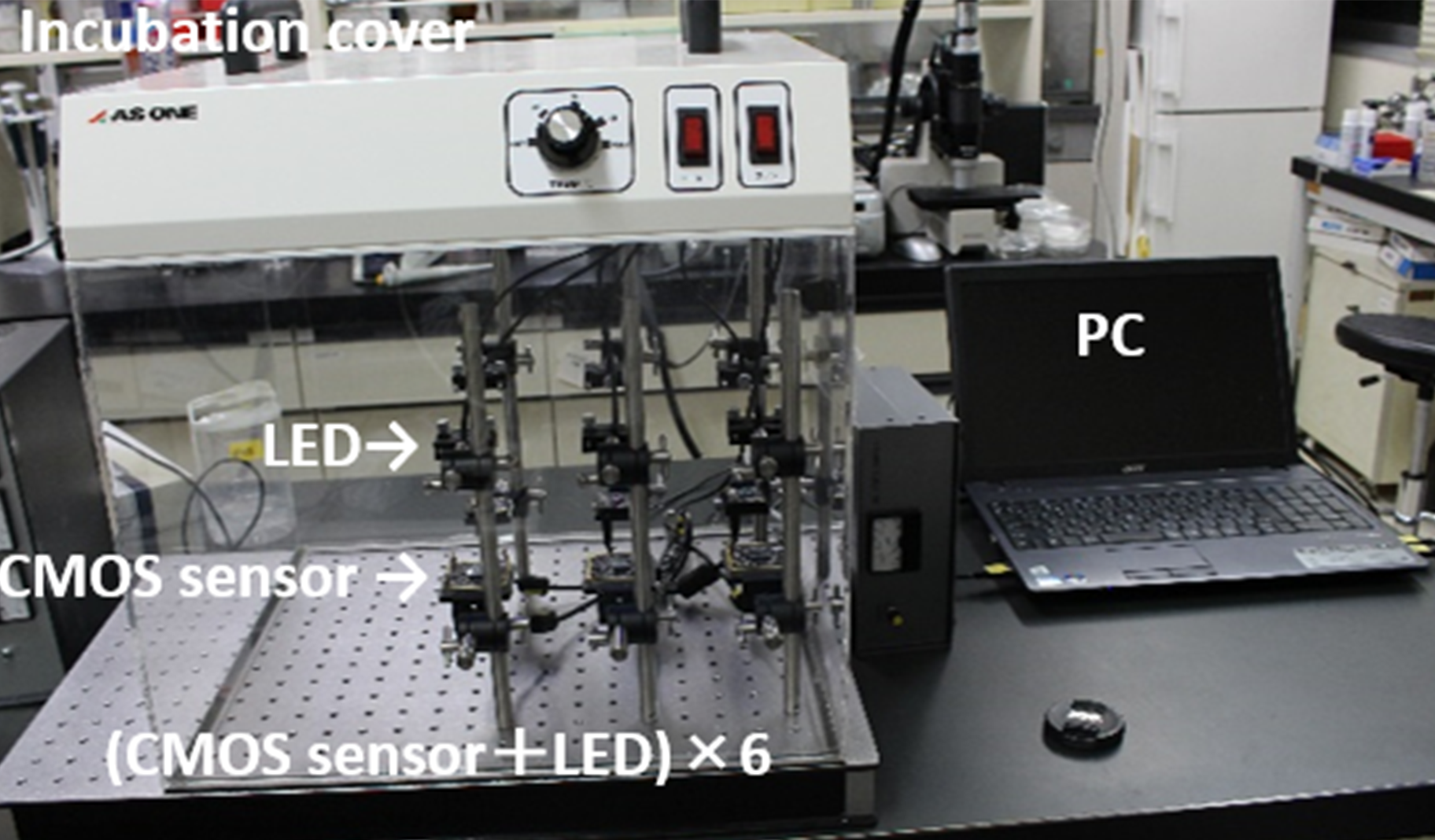

Supplement: S1 Fig — (TIF) [file pone.0174723.s001.tif]

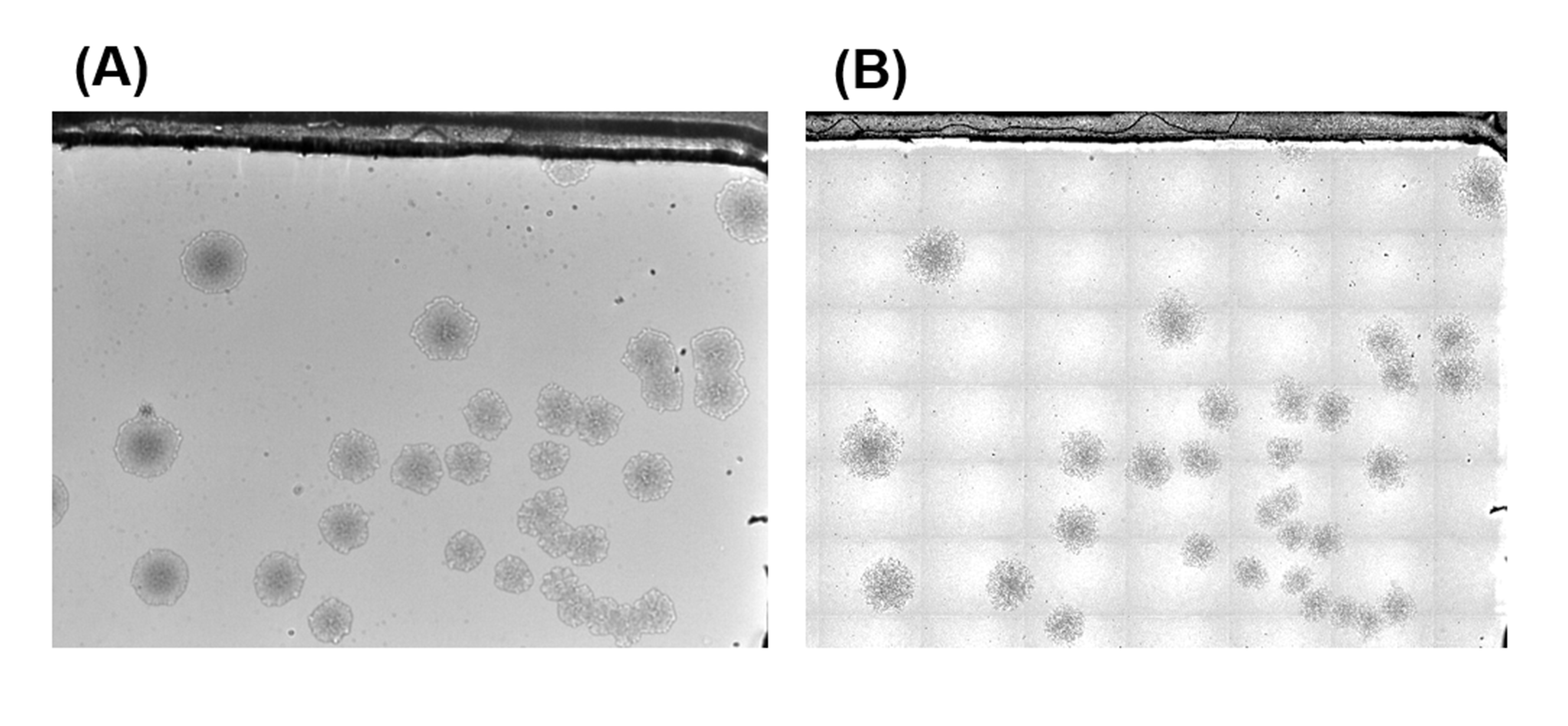

Supplement: S2 Fig — The entire image (sensing area: 6.55 × 4.92 mm) with E. coli colonies acquired by means of a CMOS sensor (A) and the corresponding microscopic image (B). (TIF) [file pone.0174723.s002.tif]

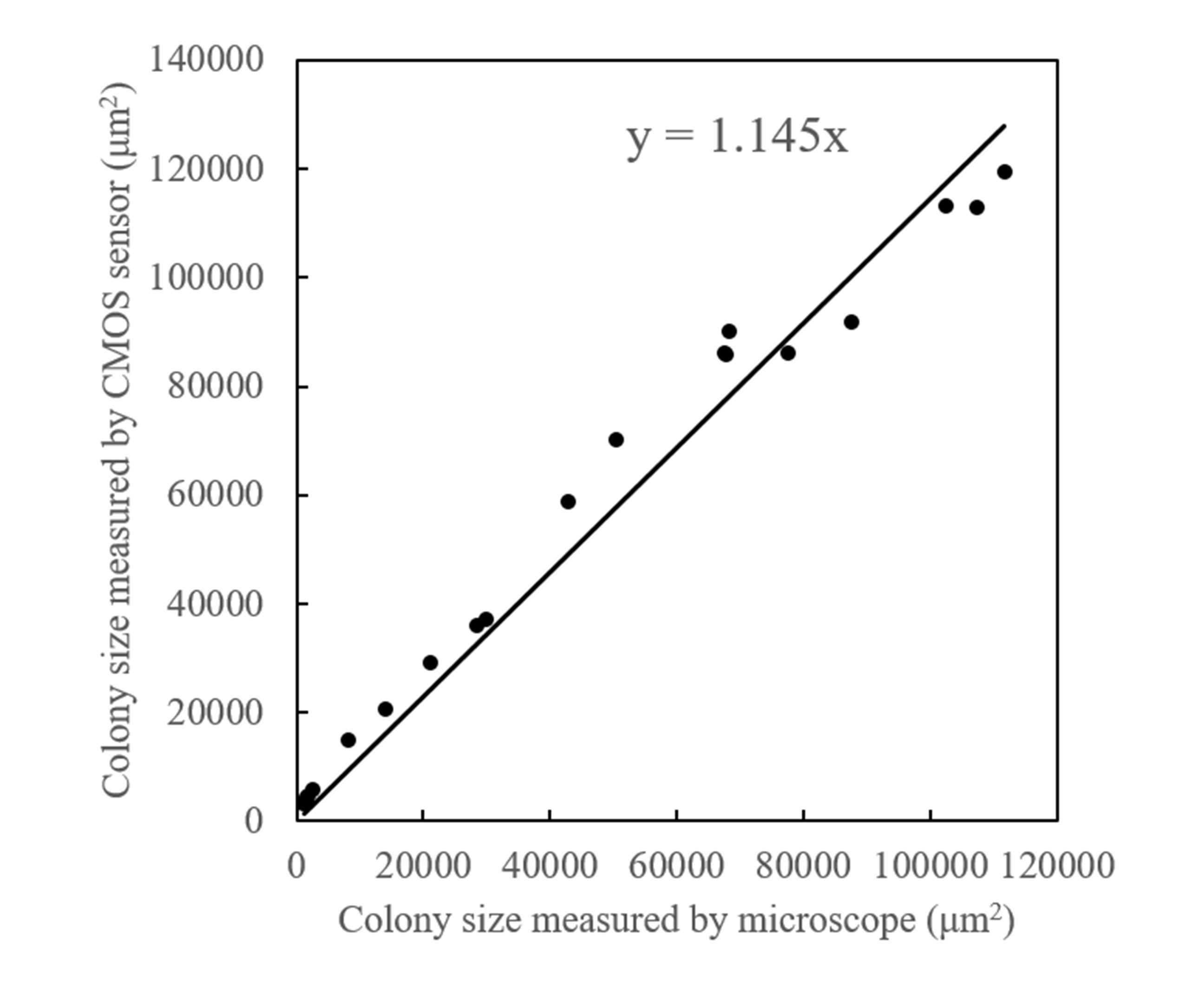

Supplement: S3 Fig — (TIF) [file pone.0174723.s003.tif]

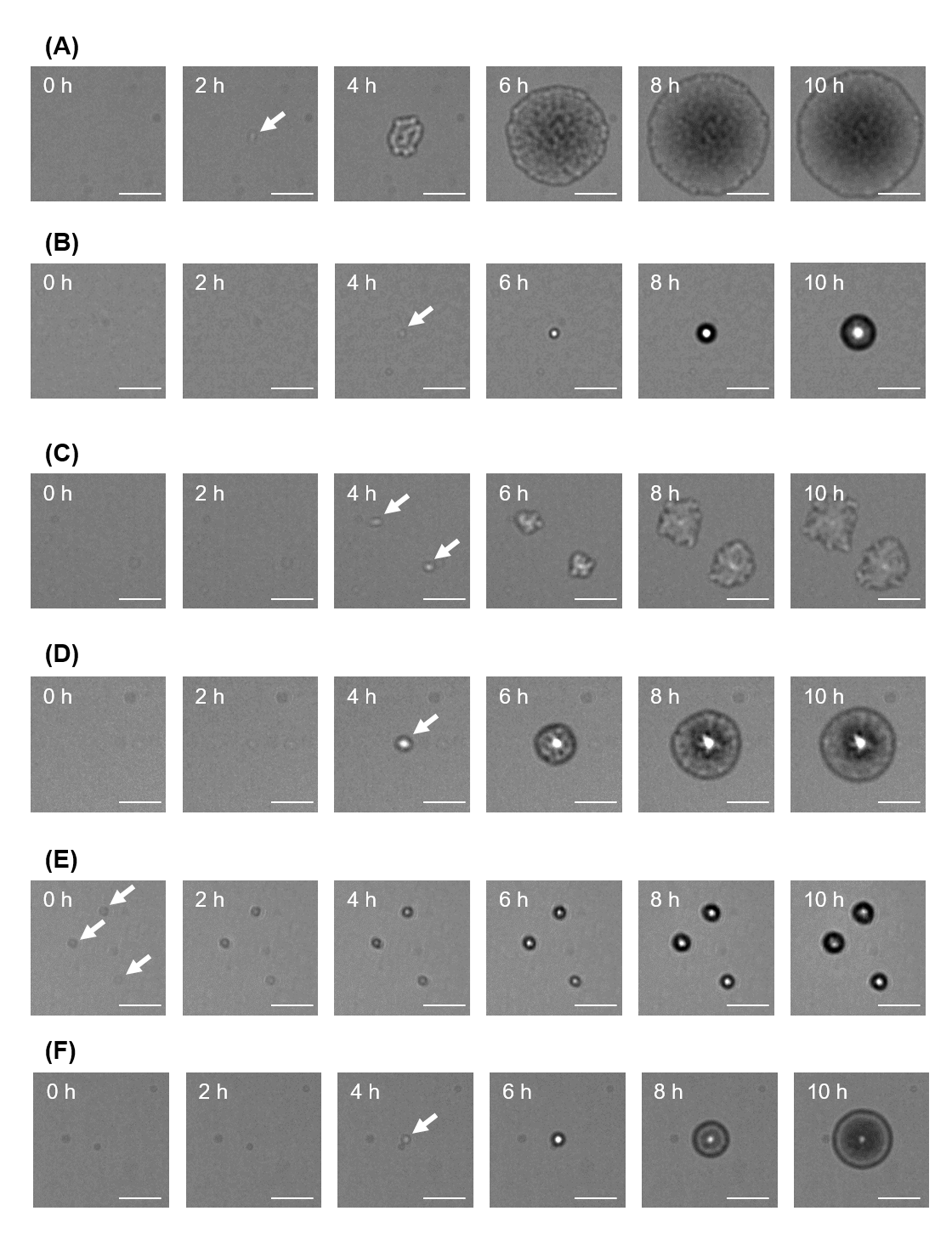

Supplement: S4 Fig — Time lapse images of colonies of (A) E. coli, (B) St. aureus, (C) P. aeruginosa, (D) Sa. enterica, (E) C. albicans, and (F) St. epidermidis acquired by the lensless imaging system. Scale bar = 100 μm. (TIF) [file pone.0174723.s004.tif]

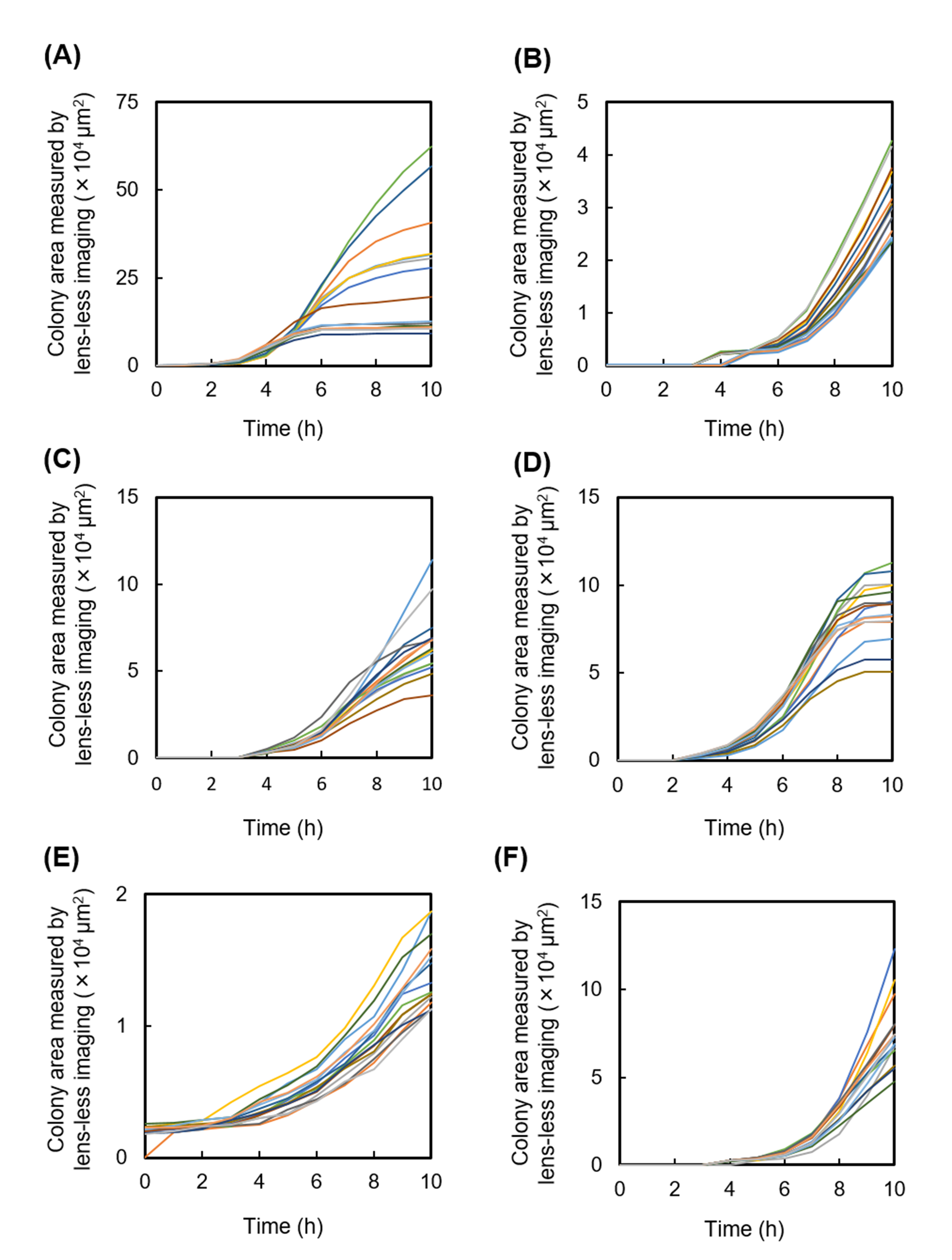

Supplement: S5 Fig — Colony size variation of (A) E. coli, (B) St. aureus, (C) P. aeruginosa, (D) Sa. enterica, (E) C. albicans, and (F) St. epidermidis measured in lensless images. Lines (arbitrary colors) are plots for individual 15 colonies. (TIF) [file pone.0174723.s005.tif]

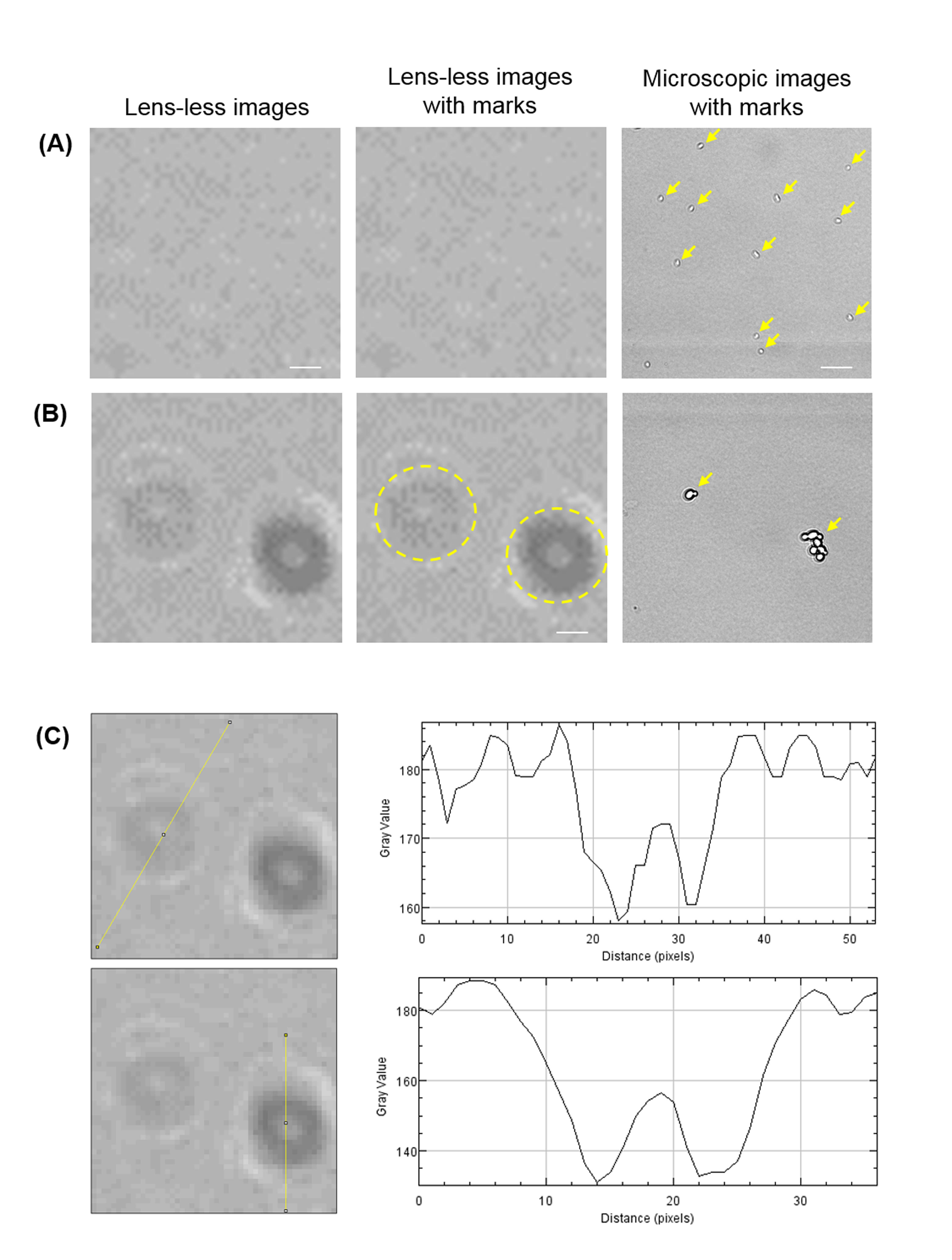

Supplement: S6 Fig — Bright-field images of E. coli (A) and C. albicans (B) on LB agar. The images were acquired by means of the lensless imaging system and microscope immediately after plating. Yellow circles on the lens-less image show C. albicans cells. Yellow arrows on the microscopic images show E. coli and C. albicans cells. Scale bar = 20 μm. Line profile analysis for the lensless image of C. albicans cells was performed (C). (TIF) [file pone.0174723.s006.tif]

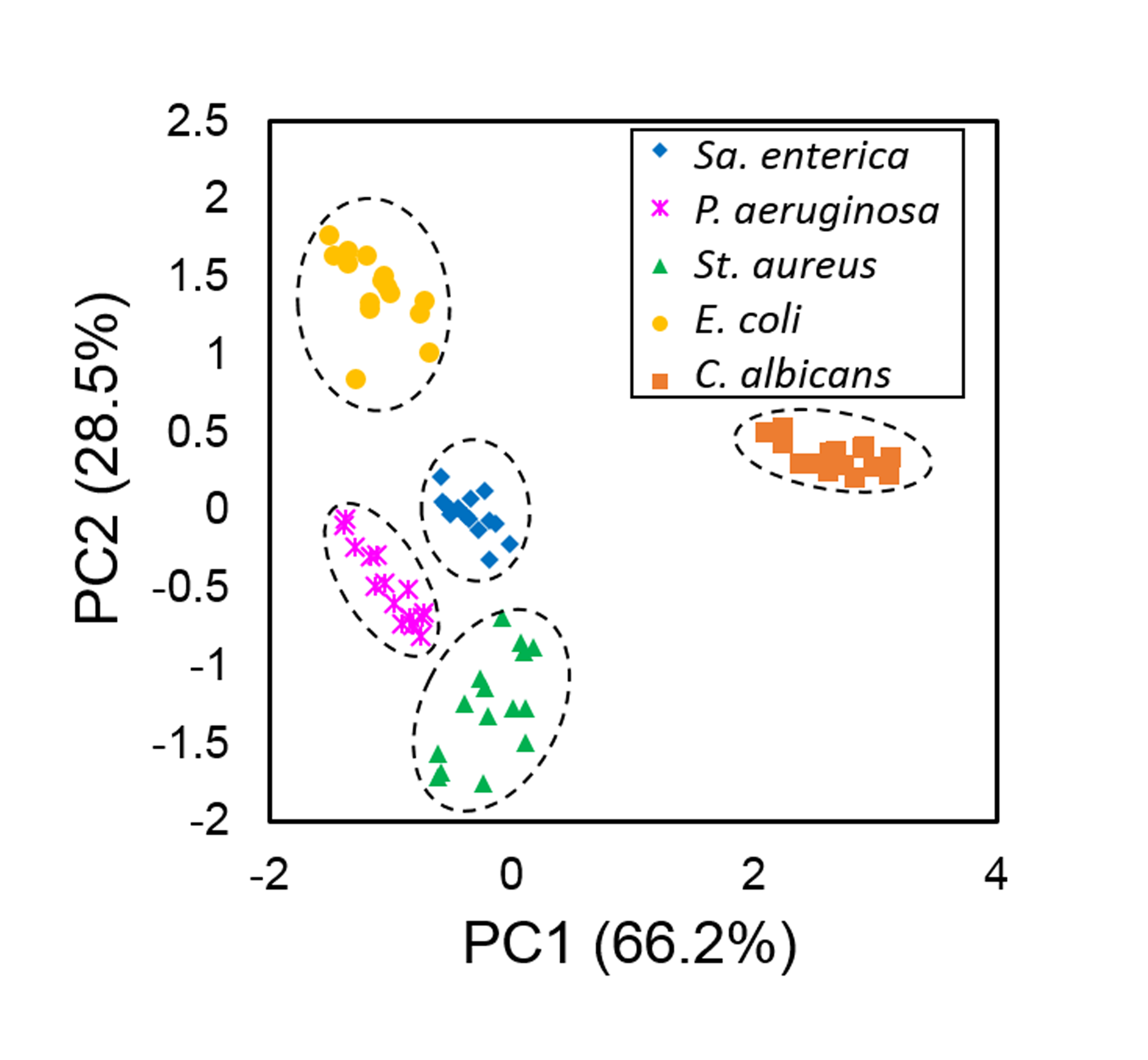

Supplement: S7 Fig — Prior to the repeated experiment, completely new bacterial cultures were prepared, and whole process including culture preparation, colony selection, image analysis was repeated by the operator different from the one who conducted the experiment shown in Fig 3. Principal component analysis (PCA) of maximum specific growth rate (μmax), colony appearance time (ta), and relative intensity (I) extracted from lensless images of 15 microcolonies of 5 microbes. Contribution ratios (%) of PC1 and PC2 are shown, respectively. Dashed circles represent the clusters generated by k-means cluster analysis. (TIF) [file pone.0174723.s007.tif]

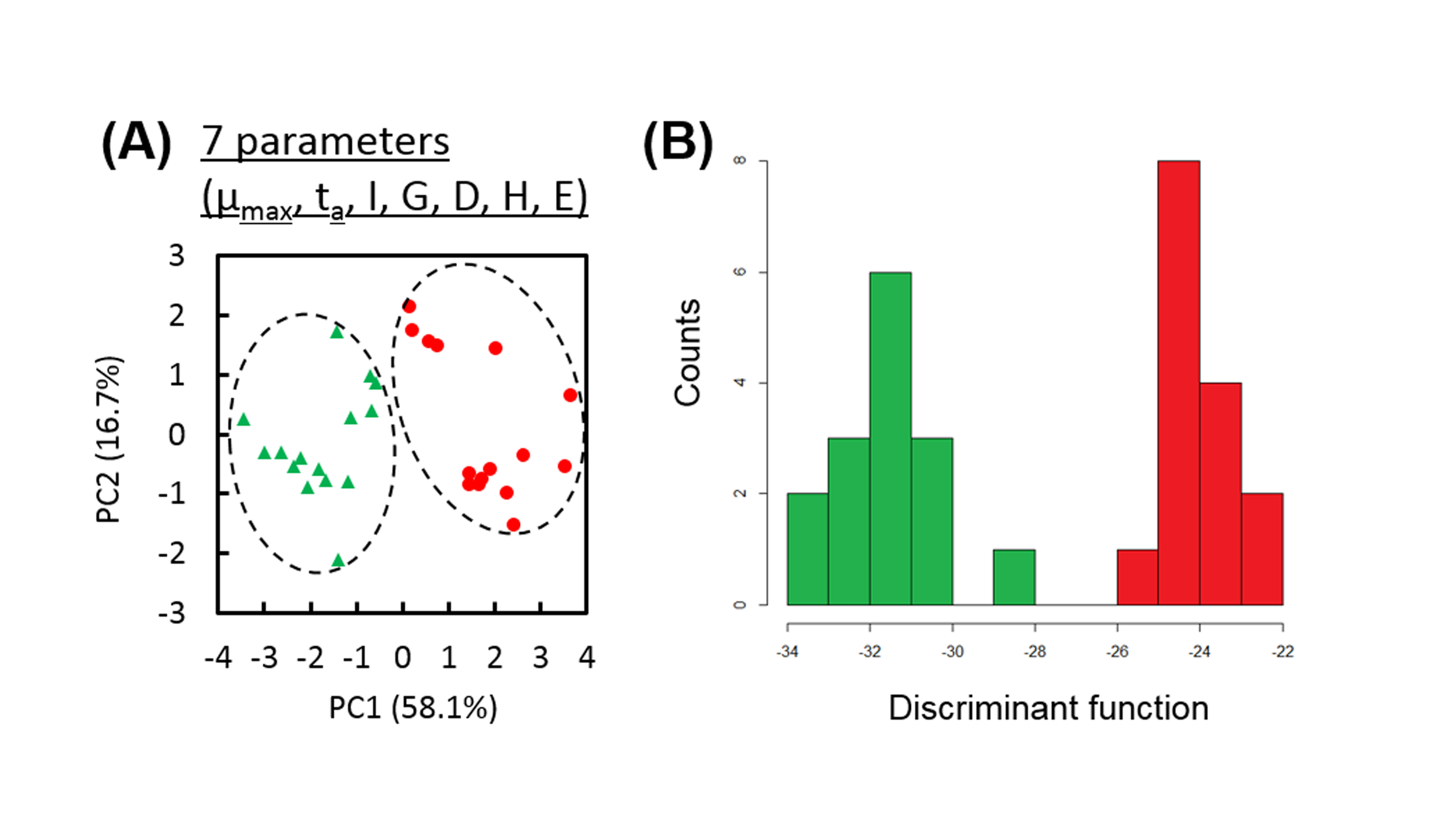

Supplement: S8 Fig — Prior to this repeated experiment, completely new bacterial cultures were prepared, and whole process including culture preparation, colony selection, and image analysis was conducted by the operator different from the one who conducted the experiment shown in Fig 4. (A) Principal component analysis (PCA) of maximum specific growth rate (μmax), colony appearance time (ta), relative intensity (I), histogram deviation (G), donutness (D), entropy (H), and energy density (E) extracted from lensless images of 15 microcolonies of St. aureus and St. epidermidis. Contribution ratios (%) of PC1 and PC2 are shown, respectively. Dashed circles represent the clusters generated by k-means cluster analysis. (B) A histogram of the value of discriminant function (F) based on linear discriminant analysis. (TIF) [file pone.0174723.s008.tif]
